# Tumor Immune Microenvironment Landscape in Glioma Identifies a Prognostic and Immunotherapeutic Signature

**DOI:** 10.3389/fcell.2021.717601

**Published:** 2021-09-28

**Authors:** Chunyu Zhang, Lirui Guo, Zhongzhou Su, Na Luo, Yinqiu Tan, Pengfei Xu, Liguo Ye, Shiao Tong, Haitao Liu, Xiaobin Li, Qianxue Chen, Daofeng Tian

**Affiliations:** ^1^Department of Neurosurgery, Wuhan University, Renmin Hospital, Wuhan, China; ^2^Department of Neurosurgery, Huzhou Central Hospital, Affiliated Central Hospital Huzhou University, Huzhou, China; ^3^Peking University China-Japan Friendship School of Clinical Medicine, Beijing, China; ^4^Department of Neurosurgery, China-Japan Friendship Hospital, Beijing, China; ^5^Department of Neurosurgery, Union Hospital, Tongji Medical College, Huazhong University of Science and Technology, Wuhan, China; ^6^Sun Yat-sen University, The Seventh Affiliated Hospital, Shenzhen, China; ^7^Department of Cardiothoracic Surgery, Jiaxing University, The First Affiliated Hospital, Jiaxing, China

**Keywords:** glioma, TIME landscape, prognosis, CIBERSORT, immune

## Abstract

The tumor immune microenvironment (TIME) has been recognized to be associated with sensitivity to immunotherapy and patient prognosis. Recent research demonstrates that assessing the TIME patterns on large-scale samples will expand insights into TIME and will provide guidance to formulate immunotherapy strategies for tumors. However, until now, thorough research has not yet been reported on the immune infiltration landscape of glioma. Herein, the CIBERSORT algorithm was used to unveil the TIME landscape of 1,975 glioma observations. Three TIME subtypes were established, and the TIMEscore was calculated by least absolute shrinkage and selection operator (LASSO)–Cox analysis. The high TIMEscore was distinguished by an elevated tumor mutation burden (TMB) and activation of immune-related biological process, such as IL6-JAK-STAT3 signaling and interferon gamma (IFN-γ) response, which may demonstrate that the patients with high TIMEscore were more sensitive to immunotherapy. Multivariate analysis revealed that the TIMEscore could strongly and independently predict the prognosis of gliomas [Chinese Glioma Genome Atlas (CGGA) cohort: hazard ratio (HR): 2.134, *p* < 0.001; Gravendeel cohort: HR: 1.872, *p* < 0.001; Kamoun cohort: HR: 1.705, *p* < 0.001; The Cancer Genome Atlas (TCGA) cohort: HR: 2.033, *p* < 0.001; the combined cohort: HR: 1.626, *p* < 0.001], and survival advantage was evident among those who received chemotherapy. Finally, we validated the performance of the signature in human tissues from Wuhan University (WHU) dataset (HR: 15.090, *p* = 0.008). Our research suggested that the TIMEscore could be applied as an effective predictor for adjuvant therapy and prognosis assessment.

## Introduction

Glioma is one of the most common malignancies in the world, with high morbidity and mortality owing to their localization and often locally invasive growth (Siegel et al., 2019). It remains the tumor with the highest incidence in the central nervous system (CNS), accounting for about 25% of primary intracranial neoplastic lesions (Weller et al., 2015; Louis et al., 2016). Meanwhile, about 50% of all gliomas with new diagnosis are classified as glioblastoma (GBM), which is the most malignant type of brain cancer (Ostrom et al., 2014). GBMs have extremely depressing prognosis, with less than 5% of observations surviving beyond 5 years when diagnosed. With the development of research, there are remarkable achievements in exploring the molecular pathogenesis of gliomas, such as the isocitrate dehydrogenase (IDH) status and O^6^-methylguanine-DNA methyltransferase promoter (MGMTp) methylation. These findings accelerate the improvement of diagnostics, classification systems, and precision therapy. However, glioma remains incurable with current clinical interventions, which traditionally include surgical resection followed by radiotherapy in combination with concurrent and maintenance temozolomide. Further investigations are essential into identification of new molecular targets, tools for prognostic assessment, and development of therapeutic regimens that provide the potentiality for improved events in the near future.

Nowadays, cancer immunotherapy by immune checkpoint blockade (ICB) has achieved great accomplishments in cancer of the bladder, head and neck squamous cell carcinoma (HNSCC), etc. (Powles et al., 2014; Garon et al., 2015; Economopoulou et al., 2016). However, recent studies have shown that the significant limitations of ICB are a large portion of the population has low or even no response to inhibitors (Rugo et al., 2018), or develop therapeutic resistance (Restifo et al., 2016), or experience severe side effects that put sand in the wheels of clinical treatments (Pitt et al., 2016). There are several important works aiming at understanding immunotherapeutic efficacy in glioma. Clinical trials of anti-PD-1 (nivolumab) immunotherapy in unselected recurrent GBM patients reveal no significant prognosis improvement, and the latest research on nivolumab in newly diagnosed GBMs has failed to show a remarkable clinical response (Chamberlain and Kim, 2017; Shen et al., 2020). These clinical results are the basis of several possibilities that GBM may have undergone such important immune reprogramming during tumor development that it has a high degree of immunosuppression and immune evasion and cannot respond to checkpoint lockouts and other immunotherapies (Sampson et al., 2020).

A growing body of research suggests that the tumor immune microenvironment (TIME) acts a critical role in tumorigenesis and treatment response (Ino et al., 2013; Shigeoka et al., 2013; Makkouk and Weiner, 2015). For example, TIME may develop several chemical and physical characteristics, beneficial to tumor progression, such as hypoxia, and increased extracellular matrix (ECM) stiffness (Barbera-Guillem et al., 2002; Bingle et al., 2002; Yu et al., 2009; Noman et al., 2014; Corbet et al., 2020). Moreover, TIME dysregulates immune effector cells resulting in suppression of immune response by recruiting cells represented by tumor-associated microglia/macrophages (TAMs), regulatory T cells (Tregs), and myeloid-derived suppressor cells (MDSCs; Badie and Schartner, 2000; Chen and Mellman, 2017; Sullivan et al., 2020). In recent years, investigations into glioma cells have indicated that CNS-resident cells or tumor infiltrative cells are inhibitory to anti-tumor immunity, such as microglia and monocytes (Grauwet and Chiocca, 2016; Varvel et al., 2016). And monocytic cells in GBM account for about 50% of the total cell counts, in comparison with <1% of cells in tumor-free brain (Badie and Schartner, 2000). Especially, anti-inflammatory M2 macrophages, polarizing from macrophages, express cytokines including interleukin 10 (IL-10), transforming growth factor (TGF)-β, and angiogenesis-associated cytokines, such as vascular endothelial growth factor (VEGF), contributing to disease progression and immune suppression (Saraiva and O’Garra, 2010; Syed, 2016; De Palma et al., 2017; Batlle and Massagué, 2019; Sawant et al., 2019).

With the advent of high-throughput technologies and advance of deconvolution algorithms such as CIBERSORT and quanTIseq (Chen et al., 2018; Finotello et al., 2019), the immune contents in the TIME could be evaluated based on RNA sequencing data. And the infiltrative fractions of immune cells calculated on CIBERSORT method have proven to be as solid as results from experimental measures such as immunohistochemistry and flow cytometry (Fu et al., 2018). This methodology has been used in cancers for exploring the association between prognosis and immunotherapy response and resistance and TIME infiltrates (Zeng et al., 2019; Fan et al., 2020; Zhang et al., 2020). However, to date, the comprehensive analysis of landscape of immune cell infiltration in glioma has not yet been completely explicated.

In the present study, CIBERSORT algorithm was employed to calculate the fractions of 22 immune cell categories based on 1,975 glioma RNA-seq profiles. Systematic correlation was calculated among the TIME infiltration patterns and clinicopathologic characteristics of glioma. As a result, we established a TIMEscore, which might act as a strong and accurate biomarker for assessment of clinical outcomes and response to immunotherapy for glioma patients.

## Materials and Methods

### Obtaining and Preprocessing Public Glioma Datasets

We methodically orderly systematically retrieved three databases -- The Cancer Genome Atlas (TCGA), Chinese Glioma Genome Atlas (CGGA), and GlioVis -- for publicly available glioma gene expression datasets. Samples with no survival information were excluded from following analysis. Totally, we obtained four datasets of 1,975 glioma samples. The microarray datasets (Gravendeel and Kamoun) generated by Affymetrix were downloaded from GlioVis database^1^ (Bowman et al., 2017). For fragments per kilobase million (FPKM), normalized gene expression data of CGGA and TCGA cohorts were obtained from CGGA database^2^ and TCGA database.^3^ RNA sequencing data with FPKM values were converted into transcripts per kilobase million (TPM) values, which were more similar to the transcripts produced by microarrays and were more comparable between samples (Wagner et al., 2012). The detailed clinical characteristics of observations enrolled are displayed in Supplementary Table 1. Sequencing data were processed and analyzed on R project (version 4.0.3). Finally, we used the “ComBat” algorithm to decrease the likelihood of batch effects as a result of non-biological technical biases among the four cohorts (Leek et al., 2012). Finally, six immune infiltrates of TCGA glioma set were obtained from TIMER webtool.^4^

### Consensus Clustering for Tumor-Infiltrating Immune Cells

R package CIBERSORT was employed to conduct quantification of the infiltration fractions of 22 immune cells in glioma samples. One thousand permutations was preset here (Chen et al., 2018), and the reference 547 gene sets (LM22 signature) were obtained from the CIBERSORT website. We applied unsupervised clustering analysis, that is, *k*-means method, into identification of TIME patterns and patient classification in the meta-dataset. This procedure was carried out by the R package ConsensuClusterPlus. Repetition of 1,000 times was preset to guarantee the stability of classification.

### Differentially Expressed Genes Related to the Tumor Immune Microenvironment Patterns

For detection of genes correlated with the TIME patterns, we grouped patients according to TIME subtypes on the basis of immune cell infiltration. Differentially expressed genes (DEGs) among the three subtypes were detected out of 12,572 genes and identified by R package limma, which performed the empirical Bayesian algorithm to calculate changes in expression levels based on *t*-test (Ritchie et al., 2015). And we applied the Benjamini–Hochberg (B-H) procedure to transform the *p*-value to false discovery rate (FDR). Genes were considered remarkably varied with FDR < 0.05 and absolute log2 fold-change > 1.0.

### Functional Annotation Analysis

Gene enrichment analysis based on the package clusterProfiler was carried out on TIME-associated DEGs (Yu et al., 2012). Gene Ontology (GO) and Kyoto Encyclopedia of Genes and Genomes (KEGG) results with a cutoff of FDR < 0.05 were considered statistically significant. Pathways between TIME groups A, B, and C were identified by conducting gene set variation analysis (GSVA) of the adjusted expression data in all glioma samples. HALLMARK gene sets from the Molecular Signatures Database (MSigDB) were downloaded and chosen as the reference set.

### Generation of TIMEscore and Survival Analysis

The flow of establishment of TIMEscore was designed as follows. First, the univariate Cox regression analysis was performed to calculate the association between the DEGs and the overall survival (OS) of patients with glioma. Then, a least absolute shrinkage and selection operator (LASSO)–Cox model with 10-fold validation was used to narrow down the prognosis-related variables and calculate coefficients of the left DEGs. Then, the expression of selective genes (*EXPi*)and their corresponding regression coefficients(*COEFi*) were used to construct TIMEscore. And the formula was as follows:

$$\text{TIMEscore}=\sum_{1}^{n}\left(E\,X\,P\,i\right)\times \left(C\,O\,E\,F\,i\right)$$

Prognostic clinical covariates such as age and IDH status of glioma patients determined based on univariate Cox regression analysis were filtered out. Finally, multivariate Cox regression analysis was employed to verify independently the prognosis-predicting feature of the TIMEscore based on R package survminer, even after being adjusted by the prognosis-associated clinical covariates.

### Human Samples

The research was approved by the Ethics Committee of Wuhan University [approval number: 2012LKSZ (010) H]. In total, 88 tissue samples from patients with glioma were acquired during surgical operation, covering 34 low-grade gliomas (LGGs) and 54 GBMs; and we designated the set as Wuhan University (WHU) dataset. The tissues were snap-frozen in liquid nitrogen and preserved for experimental purposes. All participants provided written informed consent.

### Quantitative Real-Time Polymerase Chain Reaction

Total RNA extraction was conducted by the TRIzol reagent (Invitrogen, Carlsbad, CA, United States). The PrimeScript RT Reagent Kit (RR047A; Takara, Tokyo, Japan) was employed to accomplish cDNA synthesis. RNA expression quantification was conducted on SYBR Premix Ex Taq II (RR820A; Takara, Tokyo, Japan), following the tutorials from manufacturers; and quantitative real-time (qRT-PCR) was performed on Bio-Rad CFX Manager (Bio-Rad Laboratories, Hercules, CA, United States). The 2^–ΔΔ^^Ct^ method was applied, and GAPDH was set as the reference. The primer sequences are displayed in Supplementary Table 2.

### Statistical Analysis

When comparing variables between two groups, statistical significance was calculated by the Wilcoxon test and, among more than two groups, by the Kruskal–Wallis test. Correlative degree was assessed by Spearman’s correlation method. The optimal cut points were obtained by the R package survminer to divide patients into the low- and high TIMEscore groups in each dataset for reduction of the computational batch effect. The Kaplan–Meier (K-M) method was employed to visualize the survival curves, and log-rank test to estimate the statistical significance of survival differences between the subgroups. R package forestplot was introduced to visualize the results of subgroup analysis of TIMEscore in glioma datasets. The hazard ratio (HR) in univariate analysis was calculated using the univariate Cox analysis. The independent prognosis-predicting indicators were identified by multivariate Cox regression analysis. R package survivalROC was employed to visualize receiver operating characteristic (ROC) curves and compute the area under the curve (AUC) to estimate the performance in prognostic assessment of TIMEscore at 1-, 3-, and 5-year OS and progression-free survival (PFS). All heatmaps were accomplished by the R package pheatmap. OncoPrint was employed to display mutation landscapes of TCGA glioma sets including TCGA-GBM and TCGA-LGG cohorts, conducted by R package maftools (Mayakonda et al., 2018). The chi-square test was utilized to identify the gene somatic mutation frequency differences between the TIMEscore subgroups. All statistical analyses were carried out on R project (v4.0.3). All tests were two-sided, and *p* < 0.05 was regarded as significant.

## Results

### Landscape of Glioma Tumor Immune Microenvironment

**FIGURE 1:**
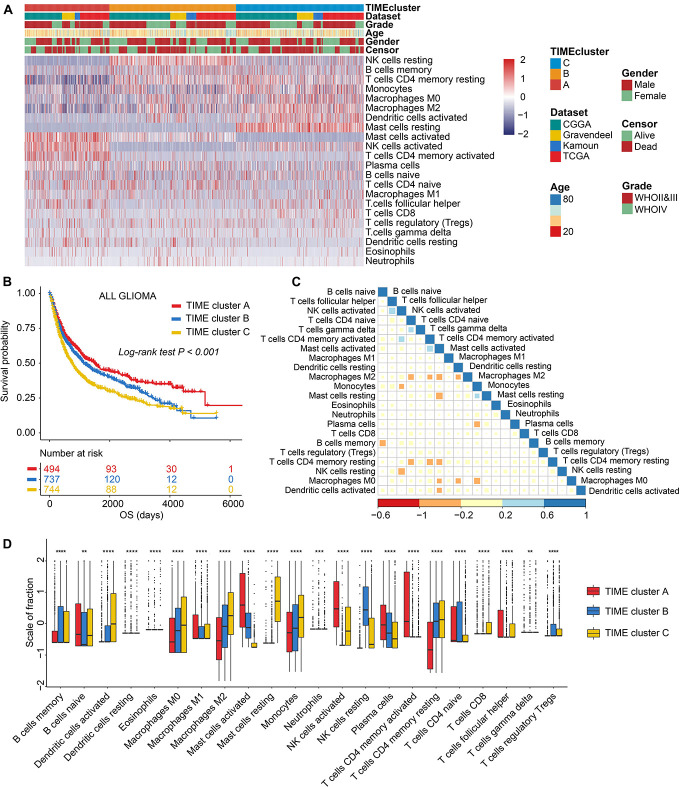
The landscape of immune cell infiltrations in the tumor immune microenvironment (TIME) of glioma. **(A)** Unsupervised cluster of tumor-infiltrating immune cells in four glioma sets. Rows refer to glioma immune infiltrates, columns samples, and colors infiltrative levels; red represents high levels and blue low levels. **(B)** Kaplan–Meier (K-M) curves for overall survival (OS) of all glioma samples grouped by TIME subtypes. **(C)** Correlation analysis of 22 immune cells. Blue represents negative correlation and red positive. **(D)** Comparing the infiltrative levels of immune cells among three TIME patterns by the Kruskal–Wallis test. ns, *p* > 0.05; *p < 0.05; **p < 0.01; ***p < 0.001; and ****p < 0.0001.

The workflow of our research is displayed in Supplementary Figure 1. First, original batch effect and batch effect removal of multiple transcriptomic data was carried out by Combat method; and results are displayed in Supplementary Figures 2A,B. Then, we performed the CIBERSORT to quantify the fractions of 22 immune cells in glioma samples (Supplementary Table 3). On the basis of 1,975 samples of the combined cohorts (Gravendeel, Kamoun, CGGA, and TCGA), unsupervised clustering was performed. We identified three independent TIME cell infiltration subtypes (Figure 1A, Supplementary Figures 2C–G, and Supplementary Table 4), and there were significant survival differences among the divided (log-rank test, *p* < 0.001; Figure 1B). Subgroup survival analysis revealed that the TIME clusters could remarkably distinguish the patients with worst prognosis in LGG subgroup (log-rank test, *p* < 0.001; Supplementary Figure 3A), yet not in GBM (log-rank test, *p* > 0.05; Supplementary Figure 3B). In addition, the correlation heatmap was drawn to display the immune cell interactions in glioma tissues (Figure 1C). To further elucidate the underlying biologic underpinnings that contribute to three distinct phenotypes, immune cell compositions among the three groups was compared (Figure 1D). TIME cluster A was identified to be related to an advantageous outcome with a median survival up to 697 days, which was characterized by high infiltrations of M1 macrophages, plasma cells activated CD4 + T memory cells, T follicular helper (Tfh) cells, activated NK cells, activated mast cells, and naive B cells, while there is a lack of infiltrations of CD8 + T cells and activated dendritic cells (aDCs). TIME cluster B experienced a median survival of 609 days, which was marked by median cell infiltration levels such as activated mast cells, and M0, M1, and M2 macrophages. The observations in TIME cluster C witnessed a worst OS, with only median survival of 504 days, and displayed the evaluated infiltration of such as immune-inhibiting cells such as monocytes, M0 macrophages, M2 macrophages, and Tregs and relatively high infiltrative levels of antitumor-involved CD8 + T cells and aDCs.

**FIGURE 2:**
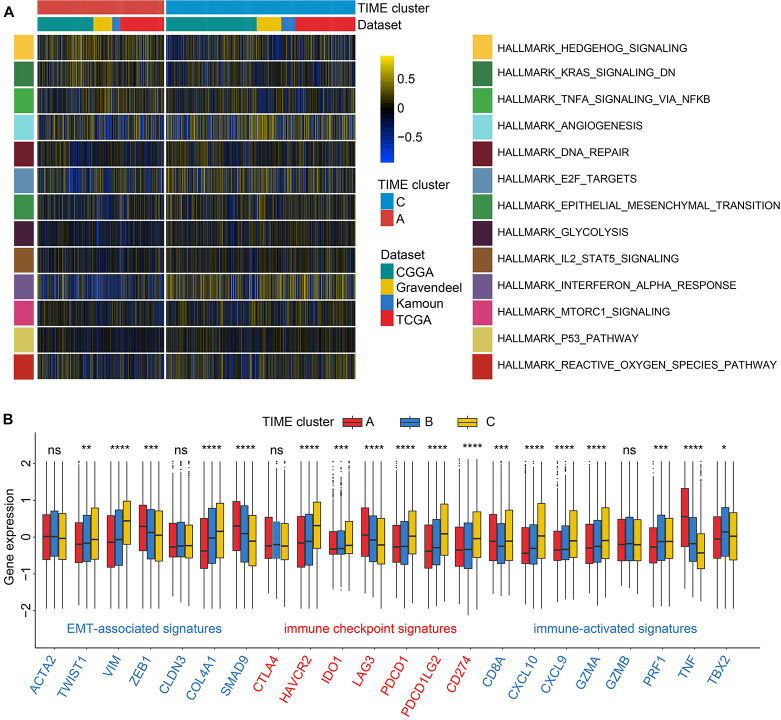
Enrichment analysis and association of transcriptome traits and TIME phenotypes in the Chinese Glioma Genome Atlas (CGGA) cohort. **(A)** Gene set variation analysis (GSVA) revealed P53 and IL2-STAT3 signaling pathways were enriched in TIME cluster C; and TNFA *via* NFκB signaling pathway and downregulation of kirsten rat sarcoma viral oncogen (KRAS) were enriched in TIME cluster A. Color represents pathway enrich scores: color blue refers to high score and yellow low. **(B)** TIME patterns were discriminated by distinct expression levels of signatures related to epithelial–mesenchymal transition (EMT), immune checkpoint, and immune activation by the Kruskal–Wallis test. ns, *p* > 0.05; *p < 0.05; **p < 0.01; ***p < 0.001; and ****p < 0.0001.

Because of the remarkable survival differences between TIME clusters A, B, and C, we, therefore, explored the biological differences using GSVA with all transcripts. As a result, we found that gene sets related to tumorigenic and immune-related processes were significantly enriched in TIME gene cluster C, including epithelial–mesenchymal transition (EMT), p53 pathway signaling, interferon alpha response, and IL6-JAK-STAT3 signaling (Figure 2A and Supplementary Table 5). Immune response-related biological process was enriched in TIME cluster A, such as tumor necrosis factor-α (TNFA) signaling *via* NFκB pathway. Pathological processes such as hypoxia and angiogenesis were also enriched in cluster B (Figure 2A and Supplementary Table 5).

Then we characterized the alterations in cytokines and chemokines levels among clusters. *CXCL10*, *CXCL9*, *GZMA*, *GZMB*, *PRF1*, *CD8A*, *IFNG*, *TBX2*, and *TNF* were regarded as immune-activated genes; IDO1, CD274, HAVCR2, PDCD1, CTLA4, LAG3, and PDCD1LG2 as immune checkpoint-associated molecules; and VIM, ACTA2, COL4A1, TGFBR2, ZEB1, CLDN3, SMAD9, and TWIST1 as EMT pathway-related molecules. TIME cluster A exhibited the high expression profiles of ZEB1, TNF, and LAG3; while GZMA, CXCL9, CXCL10, and CD8A were relatively overexpressed in TIME cluster C. EMT is a common process of paramount importance in glioma occurrence and invasion (Du et al., 2017; Brabletz et al., 2018). As depicted in Figure 2B, TIME cluster C revealed comparatively the highest expression levels of VIM, COL4A1, and TWIST1 and the lowest levels of ZEB1 and SMAD9 among the three, which is consistent with the results of GSVA step that EMT process relatively activated in TIME cluster C. And upregulated TNF in TIME cluster A might be involved in the activation of TNFA signaling *via* NFκB pathway. Cluster B revealed moderate expression levels of the above-mentioned markers (Figure 2B). Recent research has shown that neoplasms with an immune-inflamed phenotype are characterized by the existence of plentiful immune cells such as monocytic cells, which are located adjacent to the tumor core (Chen and Mellman, 2017). Meanwhile, tumor with immune-inflamed phenotype may exhibit high expression of PD-L1 in some cases. The results from the above analysis revealed that cluster C could be classified to be immune-inflamed. Cluster B was classified as immune-desert phenotype, characterized by a paucity of T cells, demonstrating the suppression of anti-tumor immunity. Cluster A was categorized into an immune-excluded phenotype, characterized by B cell-mediated adaptive immune response. In this category, recent research proposed that immune cells are located in the surrounding nest of tumor cells rather than penetrate the tumor tissues (Chen and Mellman, 2017).

### Gene Annotation and Construction of the TIMEscore

**FIGURE 3:**
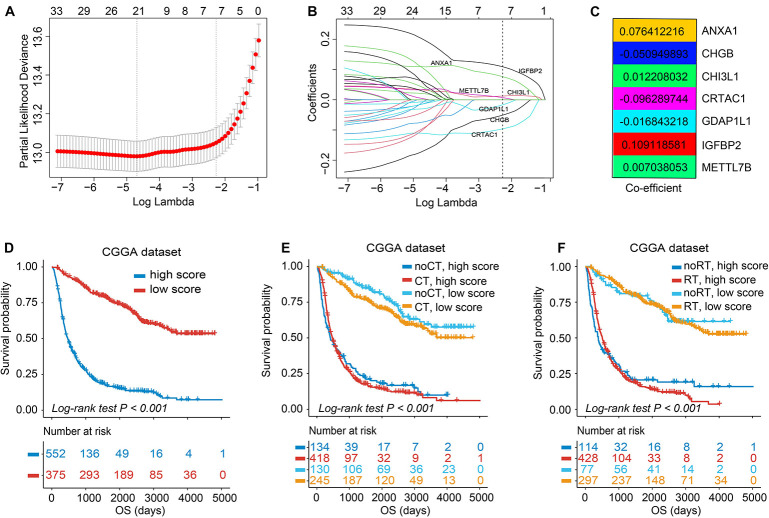
Feature selection using least absolute shrinkage and selection operator (LASSO)–Cox regression and establishment of TIMEscore. **(A)** Tuning parameter selection (λ) in the LASSO–Cox model used 10-fold cross-validation *via* minimum criteria. Dotted vertical lines were drawn at the optimal values by using the minimum criteria and the 1 standard error of the minimum criteria (the 1 – SE criteria). λ value of 0.103, with log (λ); –2.273 was chosen (1 – SE criteria) based on 10-fold cross-validation. **(B)** A coefficient profile plot of LASSO–Cox coefficient profiles of the 33 variates. **(C)** Heatmap of coefficients calculated from LASSO–Cox analysis of seven mRNAs. **(D)** K-M curve of survival differences between high and low TIMEscore groups, in CGGA sets. **(E,F)** K-M curve of survival differences between high and low TIMEscore groups when receiving adjuvant chemotherapy **(E)** and radiotherapy **(F)**.

To acquire prognosis-related quantitative indicator of TIME landscape in glioma observations, 39 DEGs calculated by the limma package were used for further analyses (Supplementary Figures 4A,B). As can be seen in Supplementary Figure 4, most genes were dysregulated between TIME clusters A and C; for example, *TNF* and *CCL4* were significantly upregulated in TIME cluster A; *FCGBP*, *IGFBP2*, and *METTL7B* in cluster C; while no gene expression significantly changed in TIME cluster B. Then functional annotation analysis revealed that in GO analysis, DEGs were mainly enriched in leukocyte differentiation, a positive regulation of neuroinflammatory response, which was considered to be related with immune regulation. KEGG analysis demonstrated that genes were enriched in modulation of Toll-like receptor, IL-17, TNF, and MAPK signaling pathway (Supplementary Table 6). In the following research, the major focus was on the CGGA set, with the most detailed and complete follow-up data. We first used univariate Cox analysis to filter out genes associated with prognosis of glioma, and there were 33 DEGs left (*p* < 0.05, Supplementary Figure 5). Then, a LASSO–Cox regression model with 10-fold cross-validation was used to contract the variables again and make the model optimized by filtering out seven genes: *METTL7B*, *IGFBP2*, *GDAP1L1*, *CRTAC1*, *CHI3L1*, *CHGB*, and *ANXA1* (Figures 3A,B). We established a signature consisting of seven mRNAs to estimate the TIMEscore for each patient, on the basis of the expression values of mRNAs multiplied by the corresponding regression coefficients (Figure 3C): TIMEscore = EXP_ANXA__1_ × 0.076412216 + EXP_CHGB_ × −0.050949893 + EXP_CHI__3__*L*__1_ × 0.012208032 + EXP_CRTA_ × −0.096289744 + EXP_GDAP__1__*L*__1_ × −0.016843218 + EXP_IGFBP__2_ × 0.109118581 + EXP_METTL__7__B_ × 0.007038053.

### The TIMEscore Predicts Glioma Prognosis

**FIGURE 4:**
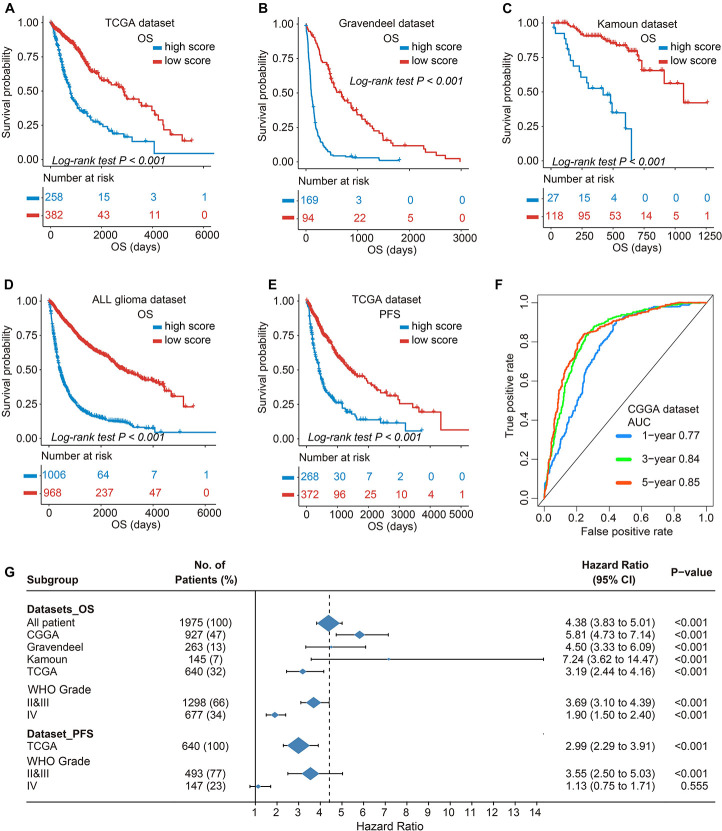
Validation of prognostic value of TIMEscore and subgroup survival analysis. **(A–E)** K-M curve of OS differences between high and low TIMEscore groups in The Cancer Genome Atlas (TCGA), Gravendeel, Kamoun, and four merged cohorts, and progression-free survival (PFS) in TCGA sets. **(F)** Receiver operating characteristic (ROC) curves estimating the predicting value of the TIMEscore in the CGGA set for OS at 1, 3, and 5 years. **(G)** Forest plot of subgroup analyses assessing prognosis-predicting value between TIMEscore groups in glioma datasets and WHO grade. Hazard ratio (HR) > 1.0 demonstrates that high TIMEscore is an unfavorable prognostic indicator.

As previously described, glioma stratification was based on the optimal cutoff calculated by the R package survminer. We separated the observations into two groups according to the cutoff value of TIMEscore. The K-M curves showed that the patients with high TIMEscore had remarkably worse OS (median OS, 506 days) in comparison with the low TIMEscore group in CGGA cohort (median OS, 1,250 days; log-rank test, *p* < 0.001; Figure 3D). Supplementary Figures 6A,B depict that patients divided into TIME cluster A had the lowest TIMEscore, cluster B the medium, and C the highest, demonstrating that a high TIMEscore was linked to TIME cluster C and a disadvantageous outcome. To be specific, we inquired into the question that whether application of adjuvant therapy disturbed the potency of the TIMEscore to predict glioma prognosis. As a result, the survival advantage was evident in patients with low TIMEscore and receiving adjuvant chemotherapy or radiotherapy (log-rank test, *p* < 0.001; Figures 3E,F). Moreover, the predicting potency of TIMEscore was proven to be solid in Gravendeel (*n* = 263), Kamoun (*n* = 145), TCGA (*n* = 640), and the total glioma cohort (*n* = 1,975; log-rank test, *p* < 0.001; Figures 4A–D). Also, the assessment of PFS in TCGA set based on the signature showed consistent results (log-rank test, *p* < 0.001, Figure 4E). The AUCs revealed that TIMEscore was a strong and accurate indicator for glioma OS at predicting 1-year survival (all cohorts: 0.75; CGGA: 0.77; Gravendeel: 0.88; Kamoun: 0.77; and TCGA: 0.69) and PFS in TCGA cohort (AUC = 0.69); at predicting 3-year survival (all cohorts: 0.78; CGGA: 0.84; Gravendeel: 0.81; Kamoun: 0.47; and TCGA: 0.75) and PFS in TCGA cohort (AUC = 0.71); and at predicting 5-year survival (all cohorts: 0.78; CGGA: 0.85; Gravendeel: 0.78; Kamoun: 0.47; and TCGA: 0.71) and PFS in TCGA cohort (AUC = 0.72). And we noticed except in the Kamoun cohort that the AUC values at predicting 3- and 5-year OS and PFS were all around 0.70, or even more than 0.70 in TCGA, Gravendeel, CGGA, and all glioma cohorts. Notably, in the Kamoun cohort, there are only two patients who survived over 3 years, so results of 3- and 5-year ROC in this cohort might be statically incorrect. The short lives might be partly caused by the human race and small-size samples in the Kamoun cohort (Figure 4F and Supplementary Figures 7A–E). In subgroup survival analysis, remarkable differences in OS between TIMEscore groups were obtained in CGGA sets (HR = 5.81, 95% CI: 4.73–7.41). Simultaneously, the prognosis-predicting value of the constructed prognostic marker was also confirmed in the other four cohorts (Gravendeel: HR = 4.50, 95% CI: 3.33–6.09; Kamoun: HR = 7.24, 95% CI: 3.62–14.47; TCGA: HR = 3.19; 95% CI: 2.44–4.16, the combined set: HR = 4.38, 95% CI: 3.83–5.01, *p* < 0.001; Figure 4G). A similar finding was also observed in TCGA_PFS cohort (HR = 2.99, 95% CI: 2.29–3.91, and *p* < 0.001). Table 1 and Supplementary Table 7 reveal that TIMEscore was detected and validated as an independent and cogent prognosis-associated indicator for OS in the CGGA, Gravendeel, TCGA, Kamoun, and combined cohort and for PFS in TCGA cohort. Finally, clinical tissues were selected for external validation, and the results demonstrated that high TIMEscore could predict the poor prognosis (log-rank test, *p* < 0.001, Supplementary Figure 8A), and ROC analysis validated the reliability of public findings (AUC: 1 year, 0.66; 3 years, 0.92; 5 years, 0.97; Supplementary Figure 8B). Meanwhile, the signature was still verified as a potent and independent marker for glioma, again in WHU set (HR = 15.09; 95% CI: 2.06–110.51, *p* = 0.008, Supplementary Figure 8C).

**TABLE 1:** Cox regression analysis of the clinical variables, and survival in the Chinese Glioma Genome Atlas (CGGA) and The Cancer Genome Atlas (TCGA) cohorts.

Covariate	CGGA_OS cohort			TCGA_OS cohort			TCGA_PFS cohort		
	HR	95% CI	*P*	HR	95% CI	*p*	HR	95% CI	*p*
**Univariate Cox regression analysis**									
TIMEscore	2.718	2.450–3.016	**<0.001**	2.184	1.877–2.543	**<0.001**	1.839	1.629–2.077	**<0.001**
Gender	1.036	0.878–1.223	0.675	1.092	0.824–1.447	0.541	1.045	0.837–1.305	0.697
Age	1.028	1.021–1.036	**<0.001**	1.074	1.062–1.086	**<0.001**	1.040	1.032–1.048	**<0.001**
Grade	4.115	3.467–4.885	**<0.001**	10.88	7.783–15.221	**<0.001**	5.598	4.392–7.135	**<0.001**
IDH status	3.039	2.560–3.608	**<0.001**	8.84	6.561–11.91	**<0.001**	6.826	5.351–8.707	**<0.001**
1p19q status	4.368	3.312–5.759	**<0.001**	3.82	2.449–5.957	**<0.001**	3.333	2.397–4.634	**<0.001**
MGMTp status	1.202	1.009–1.432	**0.039**	3.223	2.419–4.294	**<0.001**	2.856	2.241–3.639	**<0.001**
**Multivariate Cox regression analysis**									
TIMEscore	2.042	1.734–2.405	**<0.001**	1.847	1.435–2.377	**<0.001**	1.434	1.159–1.774	**0.001**
Age	1.009	1.002–1.016	**0.016**	1.053	1.039–1.067	**<0.001**	1.014	1.004–1.025	**0.006**
Grade	2.000	1.597–2.503	**<0.001**	5.020	3.065–8.223	**<0.001**	2.799	1.796–4.361	**<0.001**
IDH status	0.796	0.622–1.017	0.068	1.240	0.695–2.214	0.467	2.074	1.263–3.407	**0.004**
1p19q status	1.959	1.426–2.693	**<0.001**	1.628	0.959–2.765	0.071	1.440	0.959–2.163	0.079
MGMTp status	1.071	0.888–1.292	0.475	1.275	0.905–1.798	0.165	1.212	0.901–1.628	0.203

*HR, hazard ratio; CI, confidence interval; CGGA, Chinese Glioma Genome Atlas; TCGA, The Cancer Genome Atlas; IDH, isocitrate dehydrogenase; MGMTp, O^6^-methylguanine-DNA methyltransferase promoter.*

### Association of TIMEscore With Different Clinical Subgroups

To further inspect the predicting firmness of TIMEscore, the predictive power was calculated in patients with different gender, age, WHO grade, 1p19q codeletion status, IDH mutation status, and MGMTp methylation status in CGGA cohort.

**FIGURE 5:**
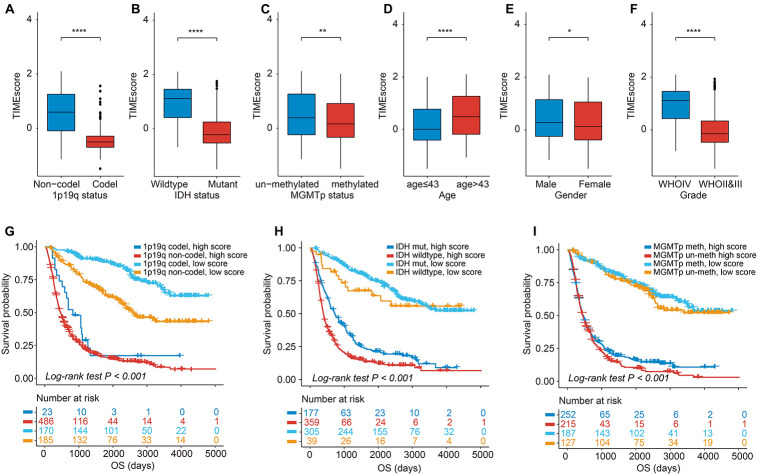
Association of TIMEscore and glioma clinicopathologic features. **(A–F)** Barplot of TIMEscore in groups with different clinicopathologic characteristics in CGGA set, compared with the Wilcoxon test. **(G–I)** K-M curves for patients with glioma in the CGGA cohort stratified by 1p19q codeletion status **(G)**, isocitrate dehydrogenase (IDH) mutation status **(H)**, O^6^-methylguanine-DNA methyltransferase (MGMT) methylation status **(I)**, and TIMEscore. *p < 0.05; **p < 0.01; ***p < 0.001; and ****p < 0.0001.

We found that except for patients with a mean age >43 years, which are associated with relatively high TIMEscore, female patients and patients with 1p19q coding deletion or IDH mutation or WHO II and III were all statistically related to low TIMEscore (Figures 5A–F). Similar consequences could be found in TCGA cohort (Supplementary Figures 9A–F). Furthermore, the K-M curves demonstrated that the disadvantageous and advantageous survival groups were still distinguished based on TIMEscore even across all clinicopathologic subgroups (log-rank test, *p* < 0.001; Figures 5G–I and Supplementary Figures 10A–C), which demonstrated that the seven-mRNA-based signature provided statistically significant OS stratification.

### Correlation of TIMEscore and Immune Infiltrates

By comparing immune cell infiltrations between the TIME groups, we found CD8 + T cells, activated NK cells, M2 macrophages, aDCs, and memory B cells remarkably infiltrated in the high TIMEscore group; and CD4 + T cells, naive T cells, activated memory CD4, and monocytes in the low TIMEscore subgroup (Supplementary Figure 11A). Then, we validated the reliability of the correlative results between TIMEscore and immune infiltrates based on six immune infiltrates of TCGA glioma set from TIMER webtool.

**FIGURE 6:**
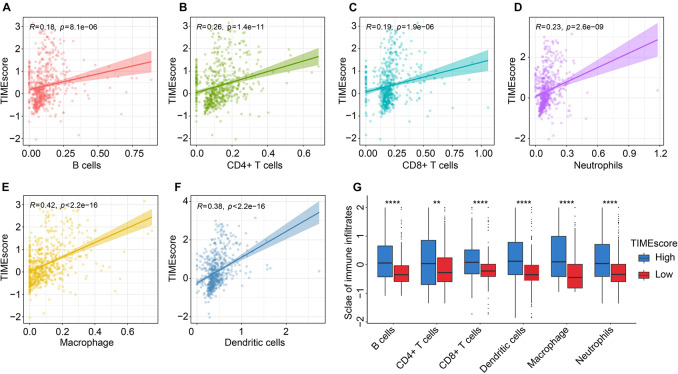
Correlation between the TIMEscore and tumor immune infiltrates using TIMER method. **(A–G)** Scatter diagrams of correlation of TIMEscore and B cells **(A)**, CD4 + T cells **(B)**, CD8 + T cells **(C)**, neutrophils **(D)**, macrophage **(E)**, and dendritic cells **(F)**. **(G)** Barplots of six immune cell infiltrative levels between high and low TIMEscore groups. *p < 0.05; **p < 0.01; ***p < 0.001; and ****p < 0.0001.

As shown in Figures 6A–F, similar consequences could be acquired through TIMER webtool. There were positive relations between TIMEscore and immune infiltrates in glioma microenvironment. Notably, TIMEscore had the strongest correlative degree with macrophage infiltration (*R* = 0.42, *p* < 2.2e−16), in comparison with others (B cells: *R* = 0.18, *p* = 8.1e−06; CD4 + T cells: *R* = 0.26, *p* = 1.4e−11; CD8 + T cells: *R* = 0.19, *p* = 1.9e−06; neutrophils: *R* = 0.23, *p* = 2.6e−09; and dendritic cells: *R* = 0.38, *p* < 2.2e−16). Meanwhile, in Figure 6G, in the high TIMEscore group, there were high infiltration levels of B cells, CD4 + T cells, CD8 + T cells, neutrophils, and dendritic cells (Wilcoxon test, *p* < 0.05). Finally, we also found that immune checkpoints such as CD274, PDCD1, and CTLA4 showed relatively high expression in high TIMEscore set (Supplementary Figure 11B). GSVA analysis between high and low TIMEscore groups revealed that immune-related biological processes such as IL6-JAK-STAT3 signaling, interferon gamma (IFN-γ) response, and inflammatory response were relatively activated in the high TIMEscore group; on the contrary, Wnt/beta-catenin pathway activation and dysregulation of kirsten rat sarcoma viral oncogen (KRAS) signaling pathway were found in the low TIMEscore group (Supplementary Table 8).

### Correlation Between Tumor Immune Microenvironment Features and Cancer Somatic Genome

**FIGURE 7:**
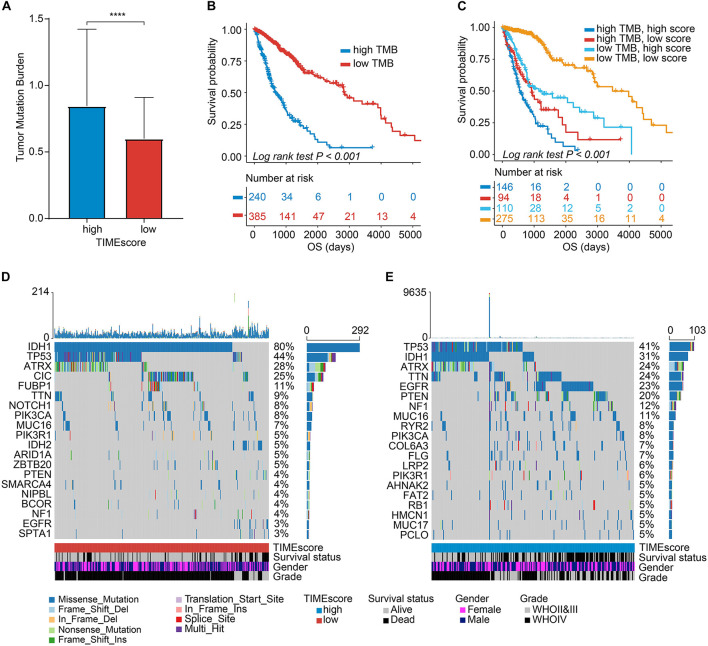
Correlation between the TIMEscore and somatic variants. **(A)** Tumor mutation burden (TMB) differences in the high and low TIMEscore subgroups. Wilcoxon test, *p* < 0.0001. **(B)** K-M curves for high and low TMB groups of TCGA glioma cohort. **(C)** K-M curves for patients in TCGA glioma cohort stratified by both TMB and TIMEscore. **(D,E)** The oncoPrint was constructed using low TIMEscore on the left **(D)** and high TIMEscore on the right **(E)**. The columns represent samples. *p < 0.05; **p < 0.01; ***p < 0.001; and ****p < 0.0001.

Recently, a great deal of research has demonstrated that tumors with an increased tumor mutation burden (TMB) in all possibility harbor the improved response to cancer immunotherapy (Nanda et al., 2016; Hellmann et al., 2018). TCGA cohort has the complete mutation files of glioma, providing solid foundations for this research. Given the important role played by TMB in clinical practice, we attempted to explore the intrinsic association between the TMB and TIMEscore. And TMB values were compared in gliomas within the high and low TIMEscore subgroups. As shown in Figure 7A, patients with high TIMEscore displayed a remarkably increased TMB, in contrast to subjects in the low TIMEscore set (Wilcoxon test, *p* < 0.001), which indicated immunotherapy might benefit gliomas with relatively high TIMEscore. Next, we used the R package survminer to obtain an optimal cutoff of TMB to categorize the gliomas into two subgroups, and there were significant survival differences after being grouped using the K-M curve (log-rank test, *p* < 0.001, Figure 7B). Considering the correlation between TMB and TIMEscore, we calculated the collaborative influence of both factors on glioma. Stratification analysis demonstrated that a seven-mRNA-based signature could still independently predict glioma prognosis, even in the presence of TMB value interference (log-rank test, *p* < 0.001, Figure 7C). Therefore, these findings demonstrated that TIMEscore could in all probability serve as a potential predicting indicator independent of TMB and an effective tool to screen beneficiaries of immunotherapy. Furthermore, we analyzed the landscape of somatic variants in TCGA glioma set between the TIMEscore subgroups by maftools. The top 20 genes with the highest alteration frequency were demonstrated in Figures 7D,E. Meanwhile, we also analyzed the gene with different alteration frequencies between the two groups. And we found that genes were significantly different such as *IDH1*, *CIC*, *EGFR*, *PTEN*, *FUBP1*, *TTN*, *NOTCH1*, *COL6A3*, *NF1*, *DNAH3*, *RYR2*, *IDH2*, *MYOCD*, *F8*, *ROS1*, *SETD2*, *LRP2*, *FBN2*, and *HCN1* (Supplementary Table 9). These findings may contribute to gain insights into glioma TIME compositions and gene mutations in immunotherapy.

## Discussion

Increasing research has demonstrated that the immune cell dysfunction within the glioma TIME boosts disorders of immune regulation and thus triggers the related malignant biological properties and poor outcomes of cancer patients. In the current study, we comprehensively analyzed landscape of interactions between the clinical features and the infiltrative cells in a meta-cohort of 1,975 glioma samples. A TIMEscore comprising seven selected mRNA features in glioma was developed by LASSO–Cox regression model. The prognostic signature developed and validated was expected to be an indicator for predicting glioma prognosis and for more effective adjuvant therapy.

Clinical trials of immunotherapy against cancer of kidney, non-small cell lung cancer (NSCLC), and HNSCC have achieved a lot (Garon et al., 2015; Motzer et al., 2015; Ferris et al., 2016), demonstrating excellent viability of the promising regimes. However, in glioma field, few studies succeed (Weller et al., 2017; Long et al., 2018; Woroniecka et al., 2018), due to the great intratumor heterogeneity and unique TIME such as dramatic T-cell malfunctions and high infiltrates of TAM (Sampson et al., 2020). In the case of the treatment strategies and prognosis assessment for glioma, the specific marker construction remains a far-reaching challenge.

The emphasis of our research was laid on the interactions of TIME with the molecular characteristics in glioma, so we first extracted immune-related genes between the TIME clusters. When analyzing functions of TIME-associated genes, our research suggested that in the GO analysis, the mRNAs were enriched in regulation of leukocyte differentiation, positive regulation of neuroinflammatory response, and mononuclear cell migration, which are considered to be related with immune regulation. In KEGG analysis, we found that the genes were significantly implicated in modulation of Toll-like receptor signaling pathway, IL-17 signaling pathway, TNF signaling pathway, and MAPK signaling pathway. These findings revealed that the DEGs between TIME clusters might play a role of significant importance in immune regulation (Iwasaki and Medzhitov, 2004; Liu et al., 2007; Veldhoen, 2017; Chen et al., 2021). For the DEGs involved in the construction of TIMEscore, METTL7B overexpression could promote glioma progression and bring an inverse prognosis to gliomas (Xiong et al., 2021); upregulated IGFBP2 in glioma has been considered as a suppressor of phosphorylation of FcγRIIB and a promoter of vasculogenic mimicry formation (Liu et al., 2019a,b); the overexpressed ANXA1 could accelerate glioma malignancy behaviors by upregulating the PI3K/Akt signaling (Wei et al., 2021); CHI3L1 and CRTAC1 have been reported to be related to glioma prognosis (Deluche et al., 2019; Xiao et al., 2020); and GDAP1L1 is expressed exclusively in human brain neuron, acting as a marker for distinguishing neuron from others such as astrocytes (Bardy et al., 2016). However, no research has been reported on GDAP1L1 in glioma.

To gain further insights into biological differences among TIME clusters, we probed into infiltrative fractions of immune cells. The results suggested that TIME cluster C had abundant infiltrations of immune-inhibitory cells, such as monocytes, M0 macrophages, M2 macrophages, and Tregs. Although high infiltration of CD8 + T cells was observed in cluster C, the cells may be in a dysfunctional state such as hyper-exhaustion (Gajewski et al., 2013; Spranger et al., 2013). Meanwhile, immune checkpoints such as PD-L1 and PD1 and proinflammatory and effector cytokines such as CXCL9 and CXCL10 can also be detected by mRNA analysis in TIME cluster C. According to recent research, TIME cluster C can be classified into immune-inflamed phenotype (Chen and Mellman, 2017). This feature reveals the existence of a preexisting anticancer immunity that could possibly be blocked by immunosuppression in the tumor bed (Rosenberg et al., 2016). Currently, response to immunotherapy most often occur in patients with inflammatory tumors, indicating potential clinical benefits from immunotherapy for these patients (Powles et al., 2014; Garon et al., 2015).

Previous research demonstrated that M2 macrophages could secrete several immune suppressors such as IL-10 and TGF-β and could downregulate IL-12 and IL-6, contributing to the suppression of T-cell activation and proliferation in tumor microenvironment (Sica et al., 2006; Qian and Pollard, 2010), as well as inducing the infiltration of Tregs (Ino et al., 2013; Shigeoka et al., 2013). Infiltration of Tregs has been recognized as a crucial mechanism in modulating immune system homeostasis and immune tolerance of the body. For example, Tregs could secrete immunosuppressive cytokines such as TGF-β, IL-10, and IL-35 (Sullivan et al., 2020); inhibit antigen presentation functions of dendritic cells and CD4 + T helper cells; and generate tumor-specific CD8 + cytotoxic T lymphocytes (CTLs), which act as barriers of anti-tumor immune response and result in the motivation of tumor immune escape. With the use of GSVA algorithm, oncogenic and immune-associated processes such as EMT, angiogenesis, IFN-γ response, and pathways such as p53 and IL6-JAK-STAT3 signaling pathways were relatively activated in TIME cluster C. The distinctive features suggested by GSVA further confirm the coexistence of slight preexisting anticancer immunity and overwhelming immunosuppressing processes in the tumor bed. Recent research reveals that CD8 + T-cell-derived IFN-γ can function as a driver and contributor of Treg fragility to boost anti-neoplasm immunity (Overacre-Delgoffe et al., 2017). In glioma TIME, IL6-JAK-STAT3 signaling pathway acts a critical role in driving tumor cell proliferation, invasion, and metastasis and negatively regulates immune response (Johnson et al., 2018). Meanwhile, research reveals that overactivation of STAT3 negatively regulates effector T cells and DCs and positively modulates infiltrations of MDSCs and Tregs. Conversely, TIME cluster A exhibited the presence of abundant immune cells such as activated NK cells, activated CD4 + T memory cells, plasma cells, and M1 macrophages, while lacking the infiltration of CD8 + T cells and aDCs. Previous research classified this type of immune-infiltrating characteristics as an immune-excluded phenotype (Salmon et al., 2012; Joyce and Fearon, 2015). For the infiltrative category, the immune cells do not penetrate the parenchyma of glioma, instead of retaining in the stroma-sounding nests of tumor cells, which makes it seem that the immune cells are actually inside the tumor (Vesely and Schreiber, 2013). Cluster B was characterized by scant activated and priming T cells and was associated with immune tolerance, corresponding to the immune-desert phenotype (Vesely and Schreiber, 2013).

Immunotherapeutic monoclonal antibodies that obstruct the PD-1 or PD-L1 could bring about durable responses in tumor patients (Mariathasan et al., 2018). With the advancement of immunotherapy in clinical trials, several studies demonstrate that TMB values and expression of immune checkpoints such as PD1 are not practical indicators to discriminate beneficiaries of immunotherapy for glioma (McGrail et al., 2021). The construction of predictors for immune checkpoint inhibitor is, therefore, encouraging. Accumulating studies support the opinions that the TIME has profound effects on glioma outcomes and on efficiency of immunotherapy. Considering the individual heterogeneity of the immune environment, we constructed and validated a scoring system that was defined as TIMEscore to quantify the TIME pattern for an individual glioma. Comprehensive analysis demonstrated that the constructed TIMEscore was an independent prognostic biomarker for glioma, and patients in the low TIMEscore group were blessed with advantageous OS and PFS, in comparison with counterparts in the high TIMEscore group. The findings were validated in TCGA, Gravendeel, and Kamoun sets; in the merged cohorts of CGGA, TCGA, Gravendeel, Kamoun datasets; and even in the clinical samples. Meanwhile, the subgroups meta-analysis further identified the strong performance of the signature in the cohorts mentioned above. By applying ROC analysis, the TIMEscore revealed the high accuracy in predicting glioma OS and PFS, demonstrating its high potentiality in clinical practice.

By analyzing the correlation between TIMEscore and clinicopathologic characteristics, we found that the signature was significantly decreased in patients with 1p19q codeletion, IDH mutant, and MGMTp methylation molecular subtype. The consequences displayed the high probability of TIMEscore applied to estimate patients’ clinical and pathological characteristics including WHO grade, IDH status, MGMTp status, 1p19q status, and TMB. 1p19q codeletion and MGMTp methylation have been confirmed to be beneficial to survival and markers for patients with high sensitivity to adjuvant chemotherapy in guiding the post-surgery treatment (Morris and Lassman, 2010; Omuro and DeAngelis, 2013). Furthermore, a recent study confirms that 1p19q codeleted WHO II and III gliomas are accompanied with decreased levels of immune infiltrates and epigenetic silencing of immune checkpoints, compared with the 1p19q non-codeletion counterparts, leading to the unsuitable immunotherapy for 1p19q codeleted LGGs (Lv et al., 2021). IDH mutation is an early event in the formation of several diffuse gliomas, which is considered to be the strongest prognostic factor for glioma (Han et al., 2020). For example, the median survival time of IDH mutant GBM is 31 months, which is more than twice the median survival time of 15 months in wild-type GBM (Yan et al., 2009), is consistent with our analysis in Figure 5B and Supplementary Figure 9B. Meanwhile, recent research reveals IDH mutation-derived D-2-hydroxyglutarate (D-2-HG) serves as a potent negative modulator for anti-tumor T-cell immunity. D-2-HG inhibits adenosine triphosphate (ATP)-dependent T-cell receptor signaling pathway, putting sand in the wheels of activating T cells in brain malignancy. Through suppressing the molecule, called signal transducer and activator of transcription 1 (STAT1), D-2-HG could result in a reduction of CD8 + T-cell immigration into the glioma region (Kohanbash et al., 2017). For WHO grading of glioma, recent research reveals the tight relations between WHO IV glioma, that is, the GBM, and immune infiltrates in that GBM has higher infiltrates of immune cells such as microglia, macrophages, and MDSCs (Komohara et al., 2008; Raychaudhuri et al., 2011), which is consistent with the findings displayed in Figures 5B, 6E that the TIMEscore positively correlated with the WHO grade and monocytic cell infiltrates.

When analyzing the correlation between TIMEscore and immune cell infiltrations, high TIMEscore was related to higher infiltrative proportion of immune cells like aDCs, CD8 + T cells, and macrophages and higher immunotherapy-related gene expression such as LAG3, IDO1, CTLA4, and PDCD1. We also observed that the patients in the high TIMEscore group demonstrated higher TMB values in TCGA glioma cohort. The above findings supported that patients in high TIMEscore might be more sensitive to immunotherapy. Although several prognostic markers have been explored in glioma field, our TIME signature based on large-scale population could be more accurate and independent in predicting the glioma prognosis, compared with indicators established (AUC: 1 year, 0.685; 3 years, 0.619; and 5 years, 0.621) (Qiu et al., 2020). Meanwhile, our comprehensive analysis revealed that the TIMEscore could also act as a potential predictor for clinical adjuvant therapy, such as radiotherapy, chemotherapy, or even immunotherapy.

## Conclusion

In conclusion, we comprehensively analyzed the TIME landscape of glioma, providing insights into the TIME infiltrative characteristics. Meanwhile, a TIMEscore system established here might boost the clinical prognosis assessment and development of treatment regimens for gliomas.

## Data Availability Statement

The datasets presented in this study can be found in online repositories: TCGA: https://portal.gdc.cancer.gov/; CGGA: http://www.cgga.org.cn/; and GLIOVIS: http://gliovis.bioinfo.cnio.es/.

## Ethics Statement

The research was approved by the Ethics Committee of Wuhan University [approval number: 2012LKSZ (010) H]. The patients/participants provided their written informed consent to participate in this study.

## Author Contributions

CZ: concept and design. CZ, HL, and NL: data collection and analysis. CZ, PX, ST, and LY: drafting the manuscript. CZ, YT, LG, ZS, and XL: revision. QC and DT: supervision for the study. All authors contributed to the article and approved the submitted version.

## Conflict of Interest

The authors declare that the research was conducted in the absence of any commercial or financial relationships that could be construed as a potential conflict of interest.

## Publisher’s Note

All claims expressed in this article are solely those of the authors and do not necessarily represent those of their affiliated organizations, or those of the publisher, the editors and the reviewers. Any product that may be evaluated in this article, or claim that may be made by its manufacturer, is not guaranteed or endorsed by the publisher.

## Footnotes

1
http://gliovis.bioinfo.cnio.es/

2
http://www.cgga.org.cn/

3
https://portal.gdc.cancer.gov/

4
https://cistrome.shinyapps.io/timer/
