# Supplementary material for: Tumor Immune Microenvironment Landscape in Glioma Identifies a Prognostic and Immunotherapeutic Signature
Source: Front Cell Dev Biol. 2021 Sep 28;9:717601. doi: 10.3389/fcell.2021.717601 (PMC8507498; doi:10.3389/fcell.2021.717601)
Supplement: Supplementary file 1 [file Data_Sheet_1.docx]

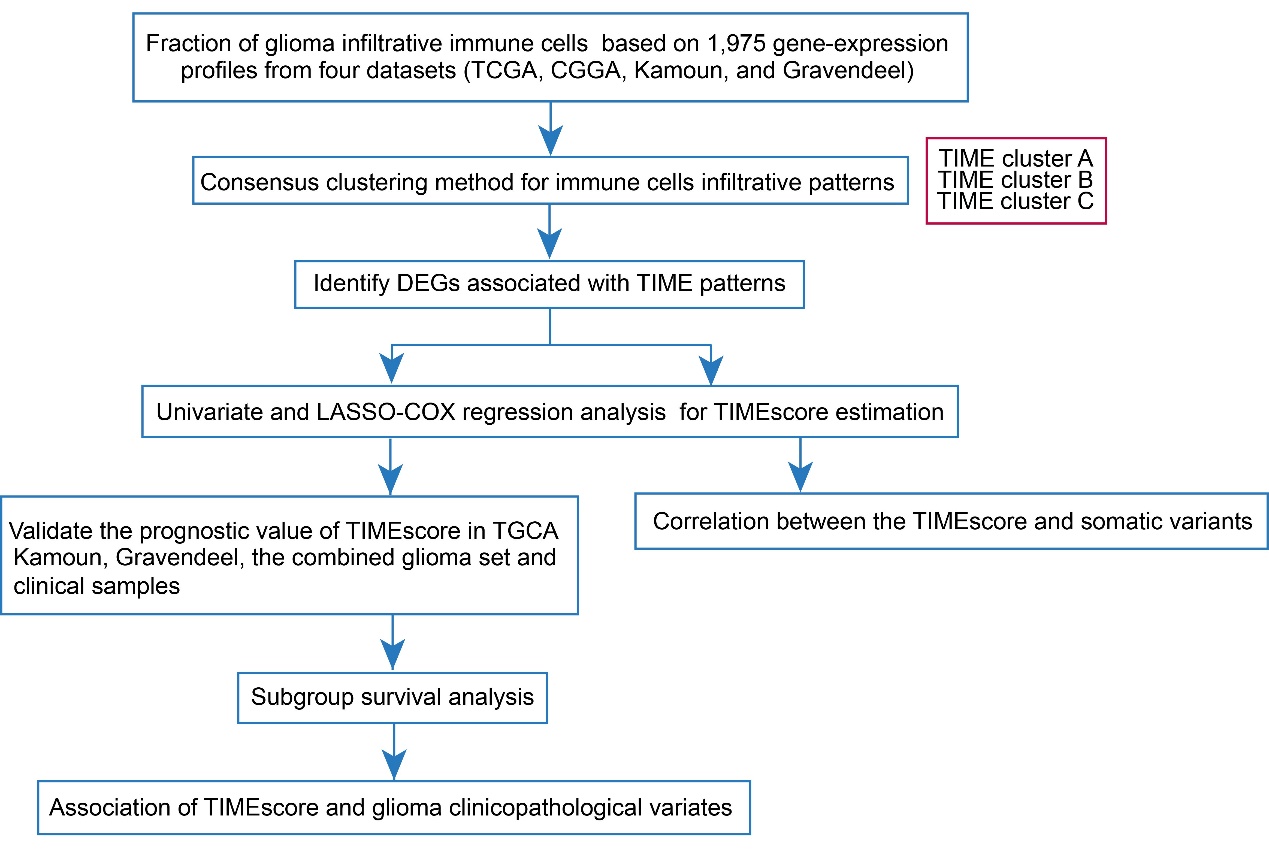


Figure S1. Overview of research flow.


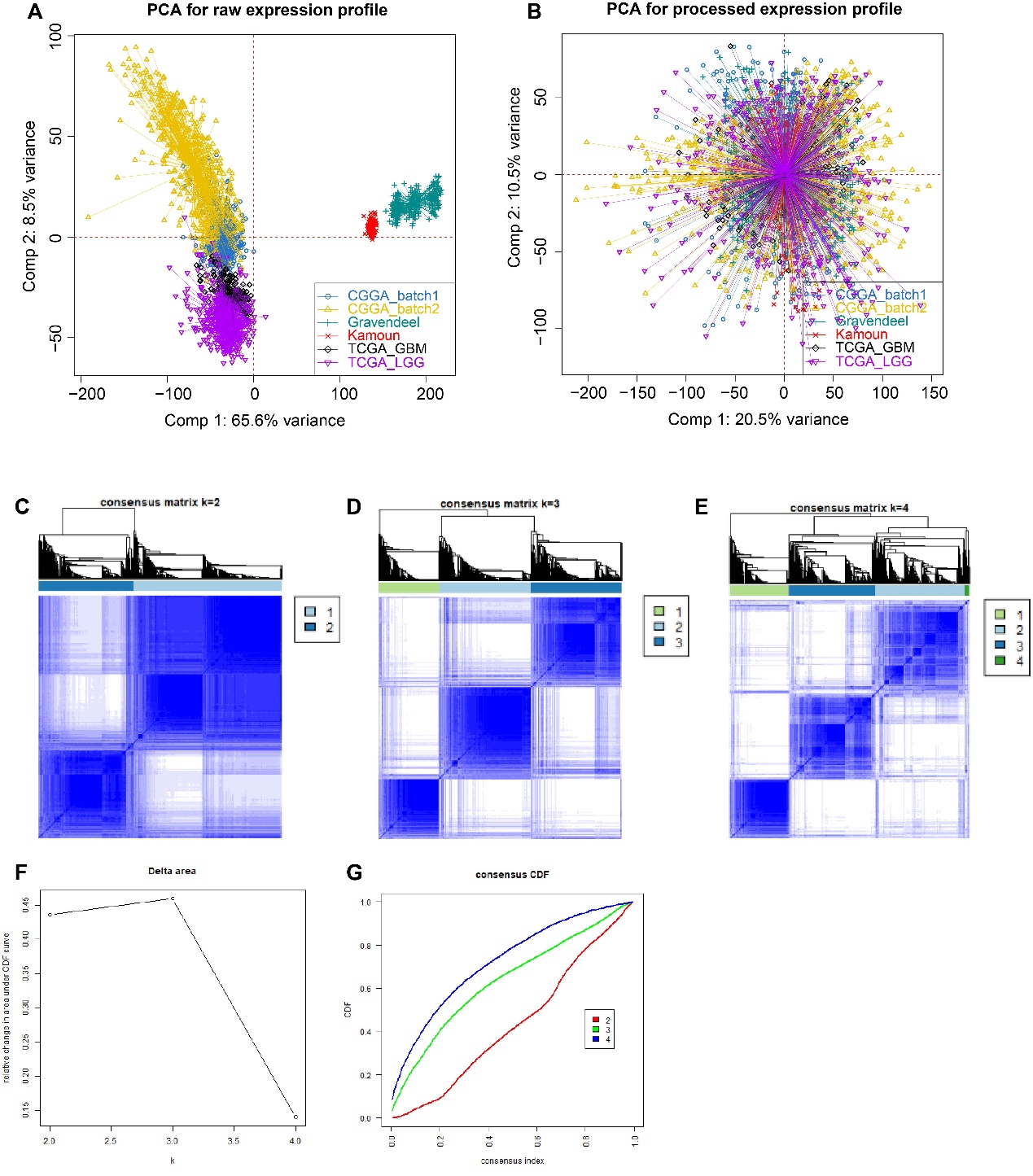


Figure S2. Batch effect of multiple transcriptomic data did exist (A) and was removed by Combat method (B). Consensus matrixes of all glioma samples for each k (k = 2-4), using 1000 iterations of hierarchical clustering for stability(C-E). The delta area (F) indicated when the k=3, there was a dramatic decrease in delta area. Cumulative distribution function (CDF) plot displayed consensus distributions for each k (G).


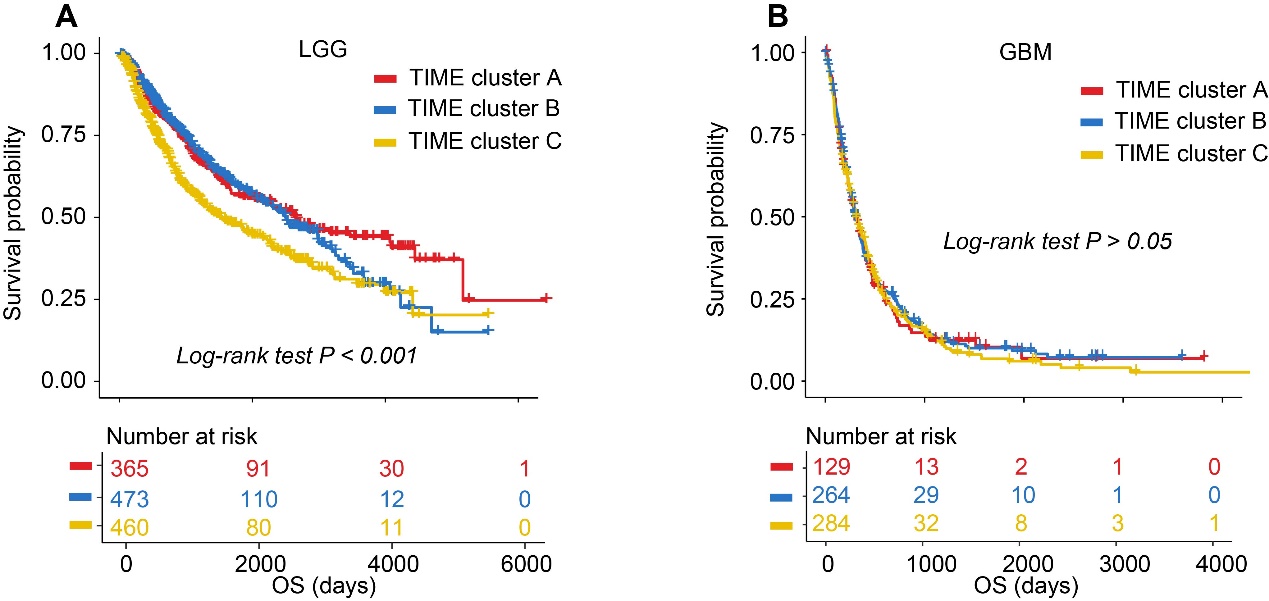


Figure S3. Subgroups survival analysis in LGGs and GBMs groups, when considered TIME cluster as a variate.


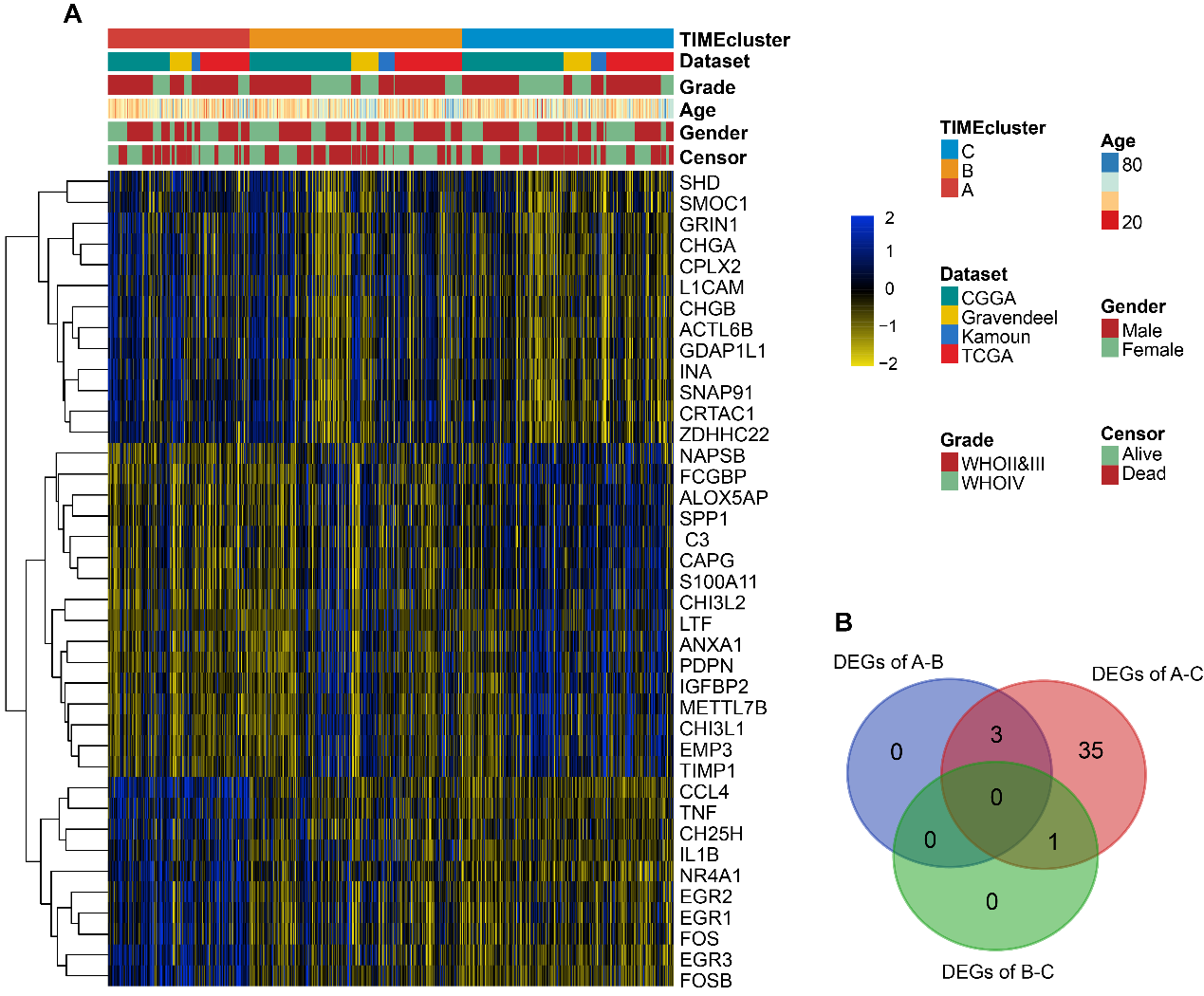


Figure S4. (A) 39 DEGs among three TIME subtypes identified by limma R package. (B) Venn diagram revealed the number of DEGs among the three clusters. Color blue represents high expression level and yellow low.


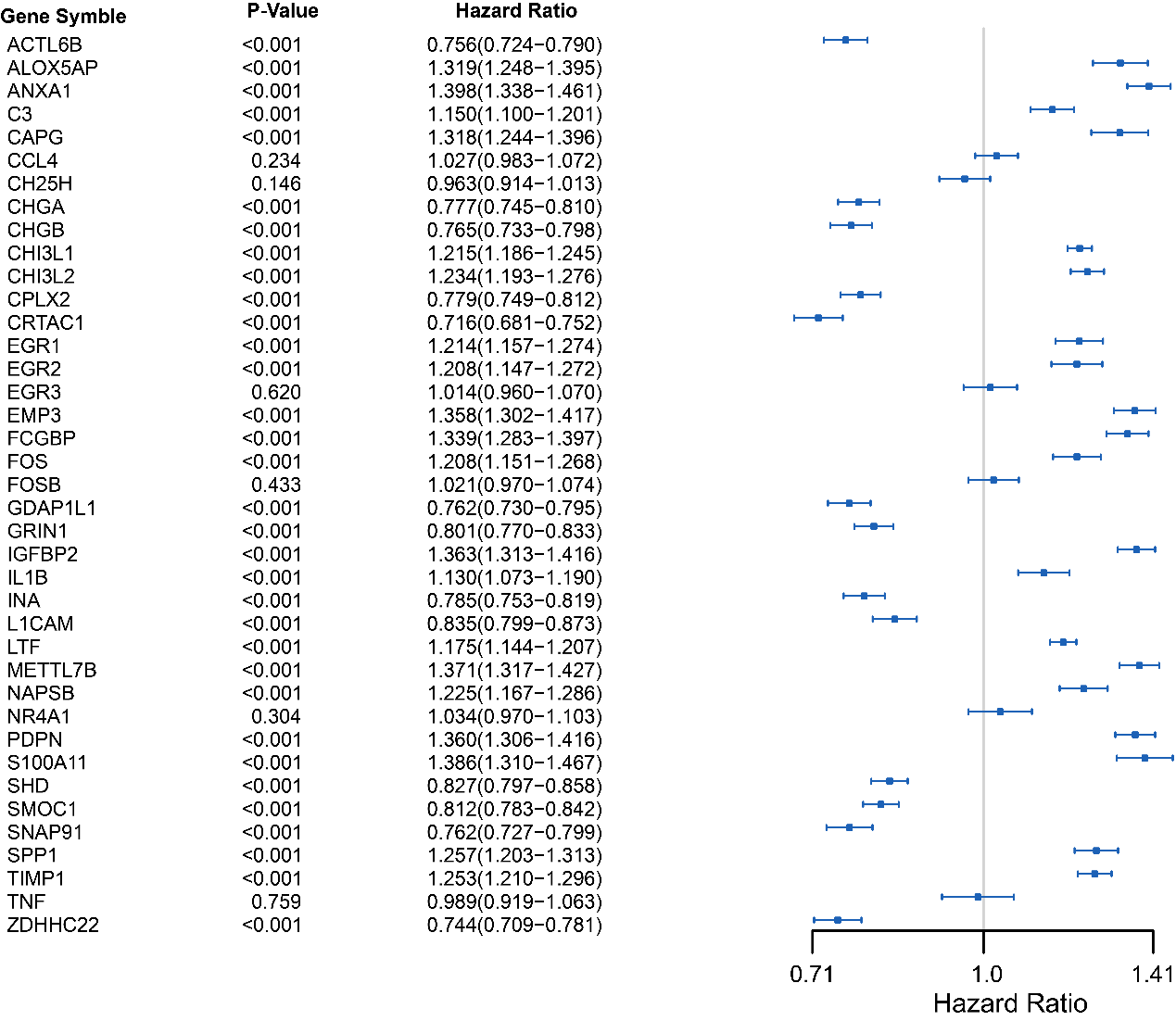


Figure S5. Univariate Cox regression analysis of 39 DEGs, and there were 33 genes were identified to be associated with prognosis of glioma.


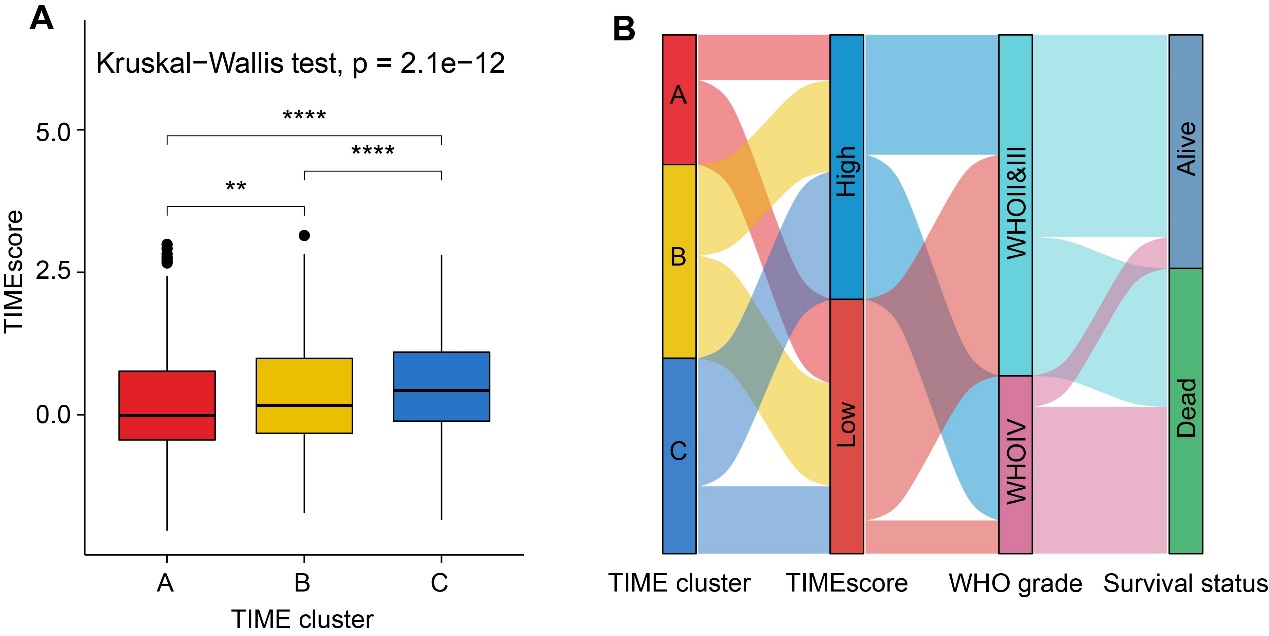


Figure S6. Relationship of TIMEscore and TIME cluster (A, and B) by Wilcoxon test. *p < 0.05; **p < 0.01; ***p < 0.001; ****p < 0.0001.


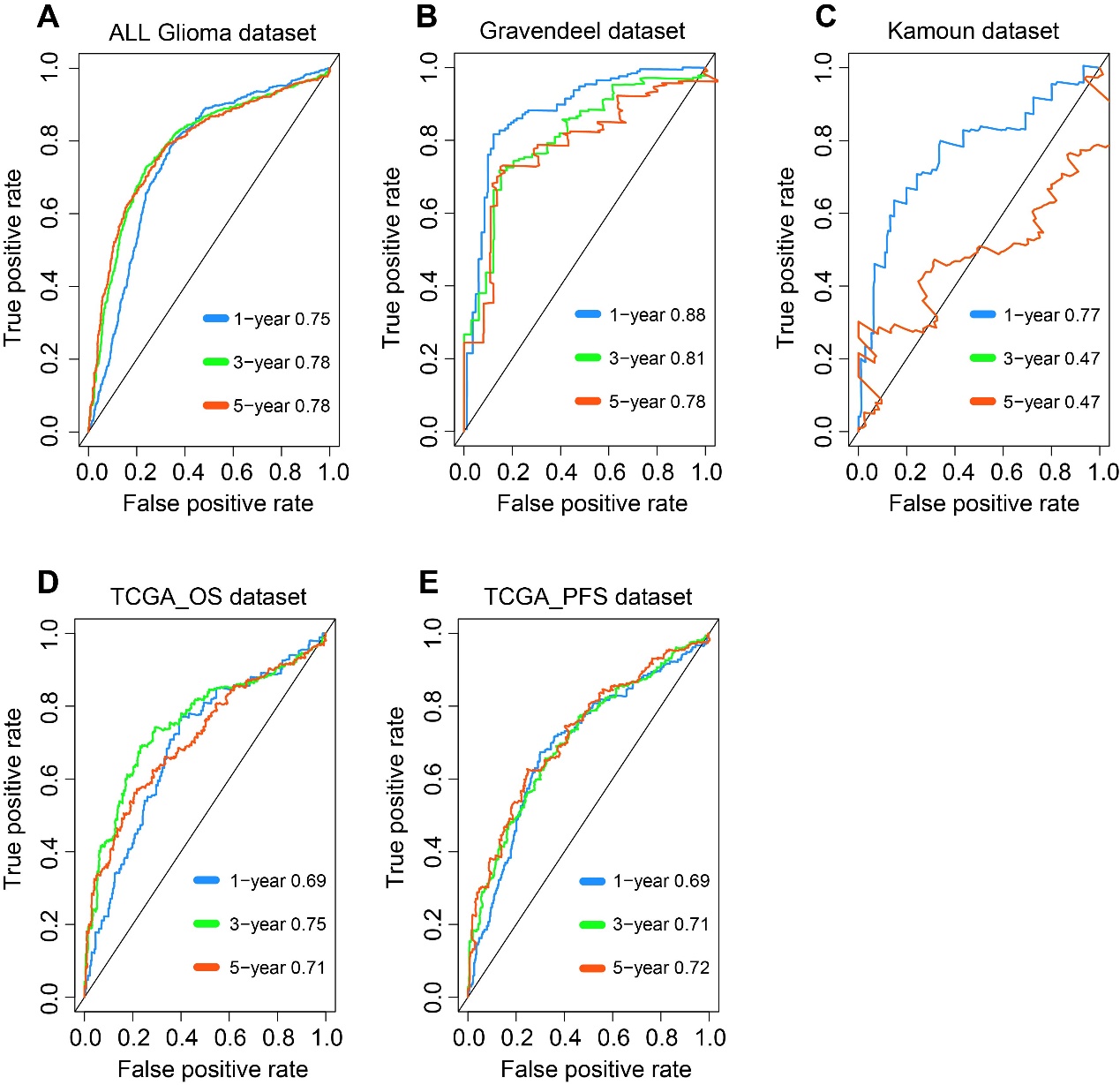


Figure S7. ROC analysis of predicting accuracy of TIMEscore at predicting 1, 3, 5-year over survival in the all glioma set (A), Gravendeel (B), Kamoun (C), TCGA (D) sets, and progression-free survival in TCGA set (E).


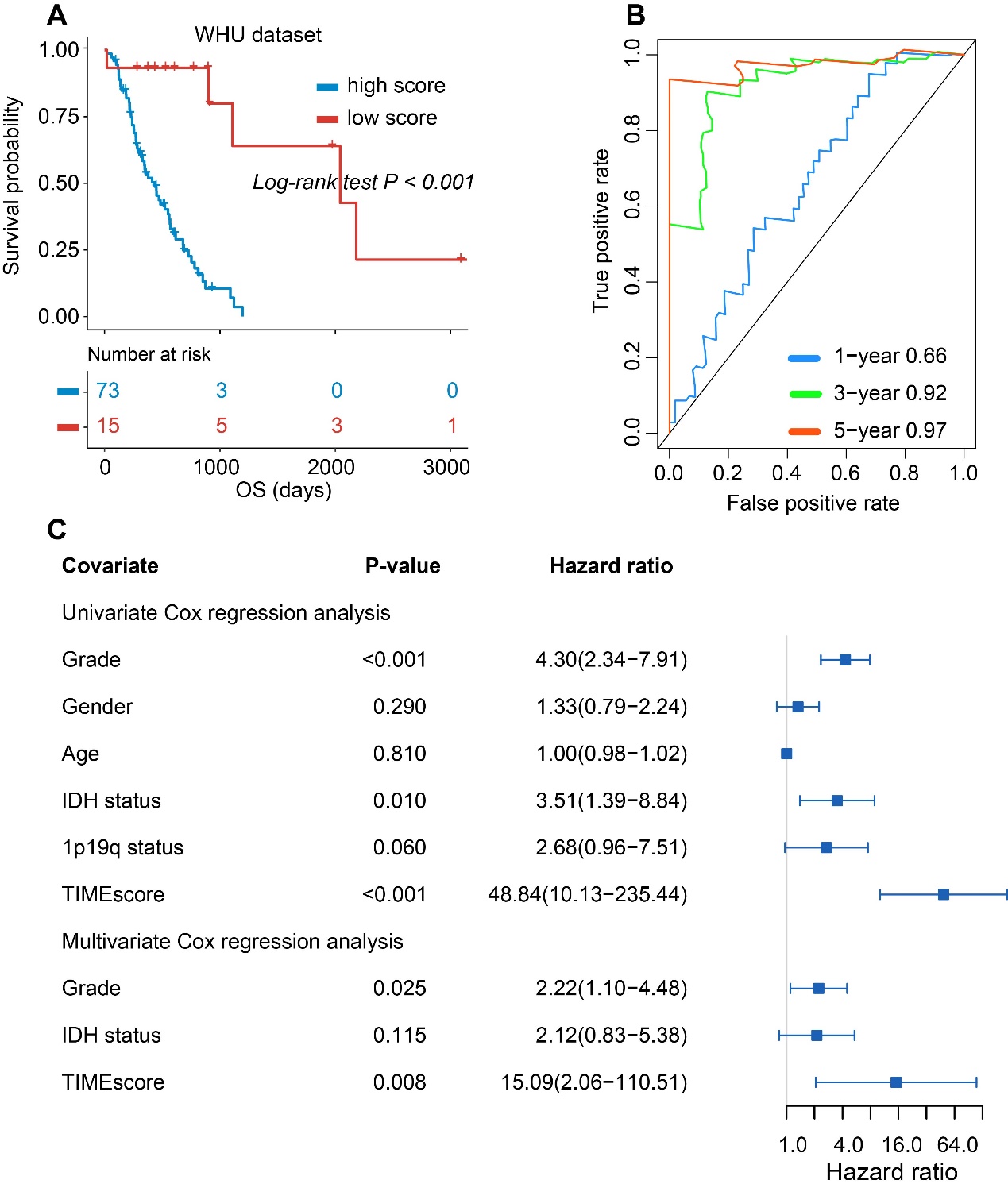


Figure S8. Validation of prognostic value of TIMEscore using 88 human tissues from WHU dataset based on Kaplan-Meier method (A), ROC analysis (B), and Cox regression analysis (C).


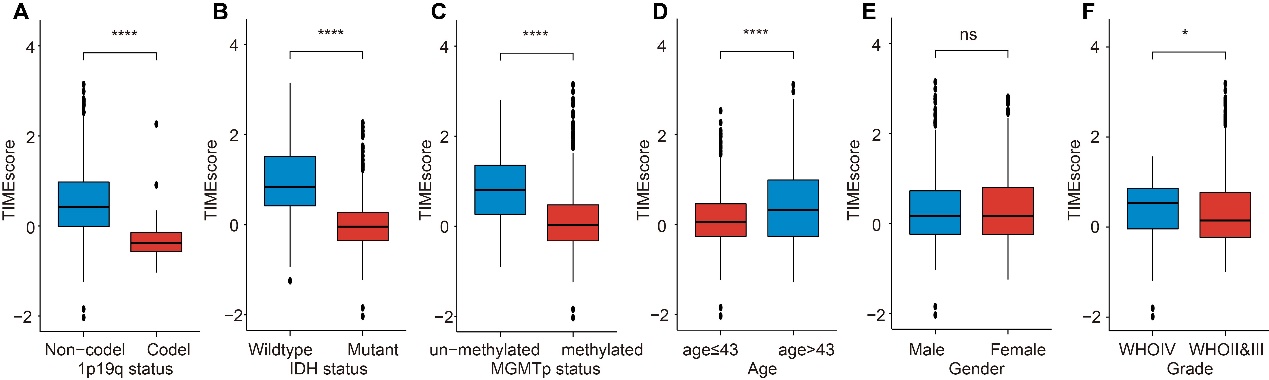


Figure S9. Barplots of correlation of TIMEscore and clinicopathological parameters of glioma by Wilcoxon test. ns, P>0.05, *p < 0.05; **p < 0.01; ***p < 0.001; ****p < 0.0001.


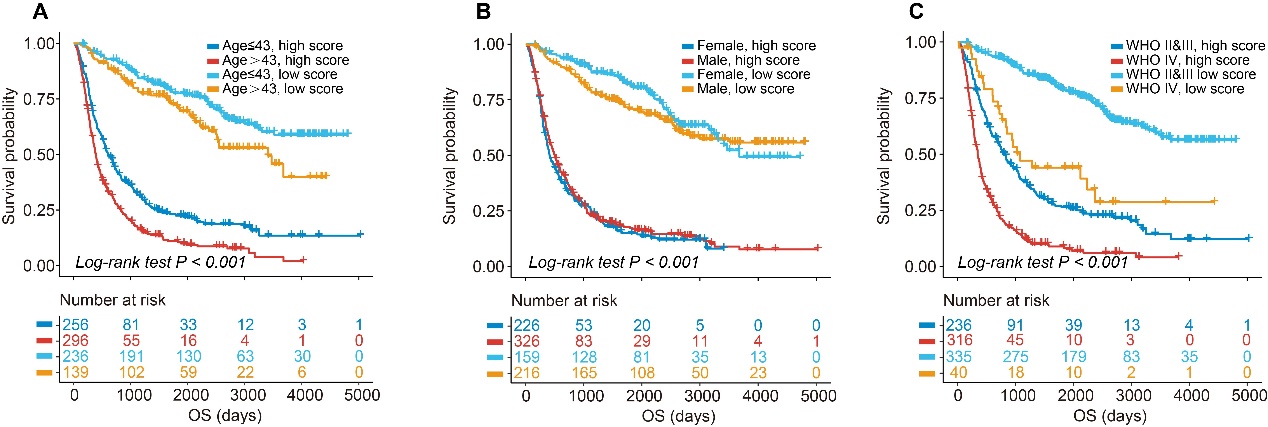


Figure S10. (A-C) Kaplan–Meier curves for patients with glioma in the CGGA cohort stratiﬁed by age(A), gender (B), WHO grade (C) and TIMEscore.


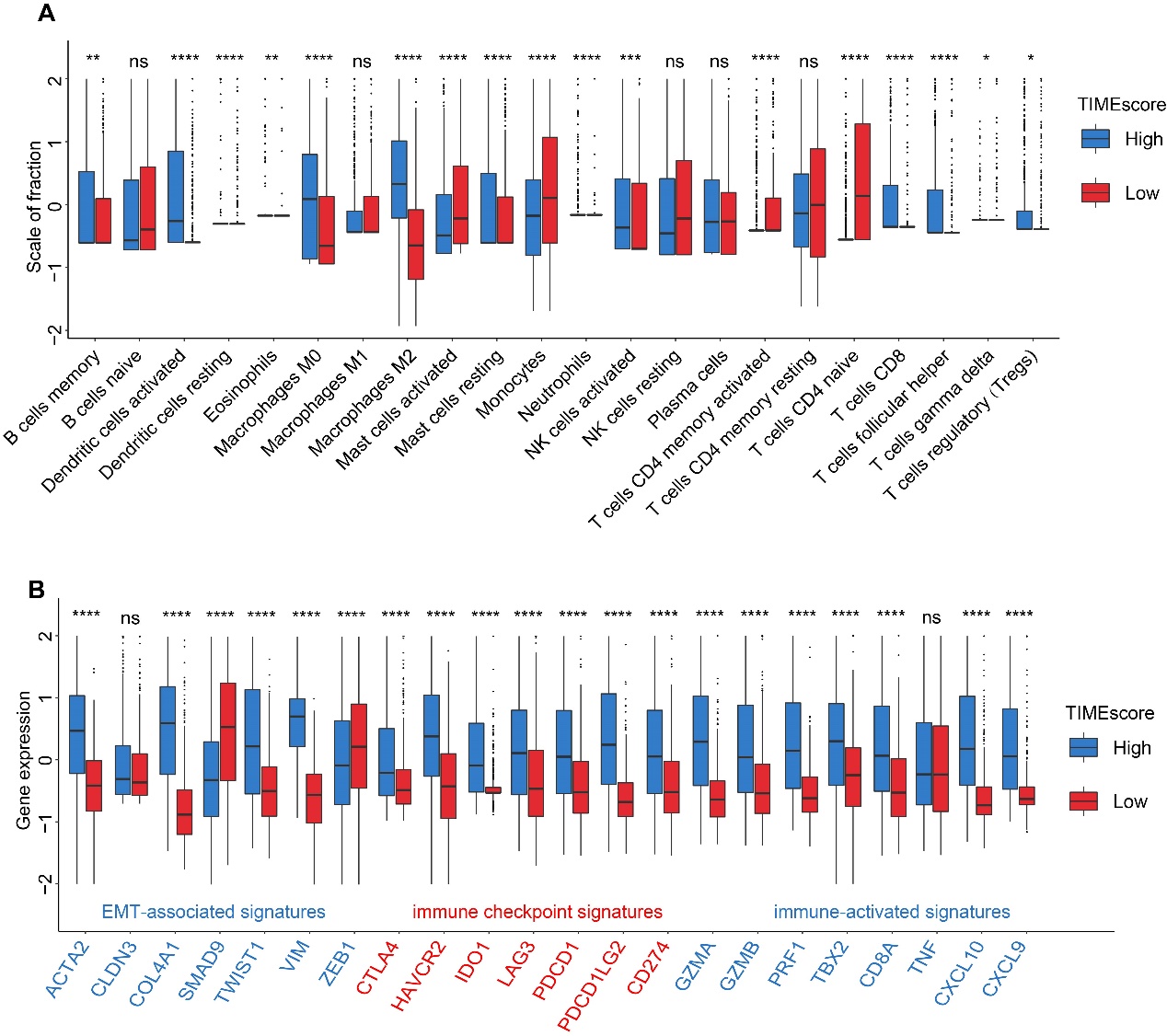


Figure S11. (A) Comparing the infiltrative levels of immune cells between TIMEscore groups by Wilcoxon test. (B) TIMEscore subgroups were distinguished by distinct expression levels of signatures related to EMT, immune checkpoint, immune activation by Wilcoxon test. ns, P>0.05, *p < 0.05; **p < 0.01; ***p < 0.001; ****p < 0.0001.
